# Genotype-phenotype correlations in recessive *RYR1*-related myopathies

**DOI:** 10.1186/1750-1172-8-117

**Published:** 2013-08-06

**Authors:** Kimberly Amburgey, Angela Bailey, Jean H Hwang, Mark A Tarnopolsky, Carsten G Bonnemann, Livija Medne, Katherine D Mathews, James Collins, Jasper R Daube, Gregory P Wellman, Brian Callaghan, Nigel F Clarke, James J Dowling

**Affiliations:** 1 Department of Pediatrics, Taubman Medical Research Institute, University of Michigan Medical Center, 5019 A. Alfred Taubman Biomedical Science Research Building, 109 Zina Pitcher Place, Ann Arbor, MI 48109-2200, USA; 2 Institute for Neuroscience and Muscle Research, Children’s Hospital at Westmead, University of Sydney, Level 3, Research Building Locked Bag 4001, Westmead, Sydney, NSW 2145, Australia; 3 Department of Neuromuscular and Neurometabolic Disease, McMaster University Medical Center, Hamilton, Ontario, Canada; 4 Neuromuscular and Neurogenetic Disorders of Childhood Section, National Institutes of Health, Bethesda, MD, USA; 5 Division of Neurology, Children’s Hospital of Philadelphia, Philadelphia, PA, USA; 6 Departments of Pediatrics and Neurology, University of Iowa, Carver College of Medicine, Iowa City, IA, USA; 7 Department of Pediatric Neurology, Cincinnati Children’s Hospital Medical Center, Cincinnati, OH, USA; 8 Department of Neurology, Mayo Foundation for Medical Education and Research, Rochester, MN, USA; 9 The Delta Pathology Group, Shreveport, LA, USA; 10 Department of Neurology, University of Michigan Medical Center, Ann Arbor, MI, USA; 11 Discipline of Paediatrics and Child Health, University of Sydney, Sydney, Australia

**Keywords:** Genotype-phenotype relationships, RYR1, Congenital myopathies

## Abstract

**Background:**

*RYR1* mutations are typically associated with core myopathies and are the most common overall cause of congenital myopathy. Dominant mutations are most often associated with central core disease and malignant hyperthermia, and genotype-phenotype patterns have emerged from the study of these mutations that have contributed to the understanding of disease pathogenesis. The recent availability of genetic testing for the entire *RYR1* coding sequence has led to a dramatic expansion in the identification of recessive mutations in core myopathies and other congenital myopathies. To date, no clear patterns have been identified in these recessive mutations, though no systematic examination has yet been performed.

**Methods:**

In this study, we investigated genotype-phenotype correlations in a large combined cohort of unpublished (n = 14) and previously reported (n = 92) recessive *RYR1* cases.

**Results:**

Overall examination of this cohort revealed nearly 50% of cases to be non-core myopathy related. Our most significant finding was that hypomorphic mutations (mutations expected to diminish RyR1 expression) were enriched in patients with severe clinical phenotypes. We also determined that hypomorphic mutations were more likely to be encountered in non-central core myopathies. With analysis of the location of non-hypomorphic mutations, we found that missense mutations were generally enriched in the MH/CCD hotspots and specifically enriched in the selectivity filter of the channel pore.

**Conclusions:**

These results support a hypothesis that loss of protein function is a key predictive disease parameter. In addition, they suggest that decreased RyR1 expression may dictate non-core related pathology though, data on protein expression was limited and should be confirmed in a larger cohort. Lastly, the results implicate abnormal ion conductance through the channel pore in the pathogenesis in recessive core myopathies. Overall, our findings represent a comprehensive analysis of genotype-phenotype associations in recessive *RYR1*-myopathies.

## Background

RyR1 is a skeletal muscle calcium release channel associated with excitation-contraction coupling [1]. The *RYR1* gene is composed of 106 exons and encodes 5,038 amino acids, making it one of the largest genes in the human genome [2]. Mutations in *RYR1* are the most common cause of congenital myopathies [3]. Both dominant and recessive mutations have been reported in *RYR1*. Dominant mutations have traditionally been associated with central core disease (CCD) and/or a susceptibility to malignant hyperthermia (MHS) [2], while recessive mutations predominate in patients with multiminicore disease (MmD), centronuclear myopathy (CNM), and congenital fiber type disproportion (CFTD) [4-6]. At this time, no specific treatments are available for any *RYR1*-related myopathy, though modifying oxidative stress may be one therapeutic avenue [7].

Until recently, the majority of research on *RYR1*-related myopathies has focused on dominant mutations in *RYR1* that lead to CCD and MHS phenotypes. Dominant mutations are enriched in three hotspots, with mutations in the N-terminus and central regions most commonly associated with MHS and mutations in the C-terminus associated with CCD [8]. Previous literature may be biased due to the fact that analysis was limited to the hotspot regions. Comprehensive studies of selected dominant mutations have led to the hypothesis that MHS associated mutations cause RyR1 hyper-excitability, while CCD associated mutations result in chronic channel dysfunction, either through excitation-contraction uncoupling or by persistent channel leakiness [9].

Much less is known about recessive mutations and their mechanism(s) of disease. Several case series have been published reporting patients with recessive mutations, though overall they have lacked sufficient patient number and power needed for more broad conclusions. The largest existing study was performed by Klein and colleagues (2012), which included 36 families with recessive inheritance. They found, as compared to patients with dominant mutations, that patients with recessive *RYR1* mutations had (1) more severe presentations with earlier onset, (2) more significant widespread weakness, and (3) more involvement of the extraocular and bulbar musculature. A smaller study from Zhou and colleagues (2007) observed that recessive *RYR1* mutations are located throughout the gene and are associated with variable histological patterns and symptoms. An additional finding, from this and from other existing studies, is that many recessive *RYR1* mutations are hypomorphic sequence changes that lead to markedly reduced or absent protein expression [1,10].

Given the growing number of cases reported with recessive *RYR1* mutations, a larger study combining and comparing these many reports is required in order to understand how various recessive mutations influence clinical phenotype, disease severity, and long term prognosis. The current study seeks to address this need by examining genotype-phenotype correlations in a cohort of 106 patients with recessive *RYR1* mutations. This cohort includes 14 previously unreported cases together with published cases from the medical literature (n = 92). We specifically analyzed whether associations exist between mutation type and location, histopathologic diagnosis, and severity of clinical features. In addition, we analyzed the distribution of recessive mutations in relation to specific domains throughout the RyR1 protein. Overall, several associations were identified, including an association between the presence of a hypomorphic allele and increased clinical severity, association of the diagnosis of CNM and/or hypomorphic alleles with ophthalmoparesis, and an enrichment of missense mutations in the MH/CCD hotspots and the pore selectivity filter. In all, this study provides a comprehensive analysis of genotype-phenotype relationships for recessive *RYR1* mutations.

## Methods

### Approvals

For the previously unreported cases, all relevant information (clinical, diagnostic, etc.) and biologic samples were obtained using a protocol approved by the IRB at the University of Michigan.

### New patient ascertainment

Clinical and diagnostic data from previously unreported cases were gathered from clinical records and from an online survey that was sent directly to colleagues or that accompanied *RYR1* genetic testing results from PreventionGenetics. Information on recessive and non-classical dominant cases (i.e. cases with dominant inheritance, but variability of symptoms due to reduced penetrance, monoallelic expression, etc.) was requested.

### RYR1 gene sequencing

*RYR1* sequencing of all coding regions was performed by PreventionGenetics using standard Sanger sequencing methods from patient genomic DNA. When possible, parental studies were performed to confirm inheritance.

### Protein expression

Levels of RyR1 protein expression were assessed by western blot analysis of frozen muscle tissue (when available) sent from participating physicians using previously published methodology [11].

### Literature review

To identify the known published cases of recessive *RYR1*-related myopathy, we searched the medical literature for all previously reported recessive *RYR1* cases and collated the clinical, histological and genetic information reported. A list of the references can be found at the end of the manuscript [1,2,4-6,9,10,12-33] and in Additional file 1: Table S1.

### Basis for genotype-phenotype assessment

We compiled the following information: *RYR1* mutations, parental testing results, family history, features of muscle biopsy, RyR1 protein expression, clinical features, age of onset, severity of weakness, motor function, respiratory function, and clinical/pathologic diagnosis. Cases with insufficient clinical or diagnostic information were excluded from the subsequent group analyses. Cases were clustered into core myopathies (MmCD, CCD, and other core myopathies), CNM and CNM-like myopathies, CFTD and other patterns. See Additional file 2: Table S2 for full descriptions.

### Disease severity ratings

A disease severity rating scale (Additional file 3: Table S3) was created for this study to investigate whether there are relationships between mutation type/position, presence of ophthalmoparesis, histopathologic diagnosis and disease severity. Ambulatory status and respiratory status were used in this rating scale.

### Stratification of recessive RYR1 mutations

Recessive mutations were divided into two groups based on *in-silico* analysis. In the first group, we included all mutations that were predicted to abolish or markedly decrease RyR1 protein production as hypomorphic alleles. These included nonsense mutations, frame-shift mutations, and splice mutations that lead to, or are predicted to cause, reduced levels of the mRNA transcript. The second group of recessive mutations included missense and small in-frame indels (insertions/deletions) which would likely result in approximately full-length but functionally abnormal RyR1 protein.

### Analysis of recessive RYR1 mutations

We initially compared mutation type (missense/indels vs. hypomorphic alleles) with level of RyR1 protein expression (when available), histological diagnosis, clinical severity, and whether or not ophthalmoplegia was present using Chi-squared or Fisher’s Exact tests. We then investigated whether the position of missense and small in-frame indel mutations (i.e. mutations that are likely to be incorporated into expressed RyR1 channels) correlates with clinical or histological features. We excluded hypomorphic mutations from this analysis since the position of these mutations in the *RYR1* gene sequence is predicted to have little relationship with RyR1 function domains. Information about amino acid sequences that contribute to functional domains in *RYR1* was obtained from our recent review of the literature [34]. For a list of functional domains and their amino acid sequences please see Additional file 4: Table S4. For the analysis of functional domains, recurrent mutations that occur in multiple individuals or families were only counted once (the most frequent clinical or histological phenotype associated with each mutation was used) to avoid skewing the results. The observed percentages of mutations residing in each domain and their 95% or 99% Wilson confidence intervals were calculated using SAS 9.2 software.

## Results

### A newly identified cohort of 29 families with RYR1-related myopathies

We identified 29 new cases (14 with recessive inheritance from 12 families and 15 with dominant inheritance from 14 families) with *RYR1* mutations (Additional file 5: Table S5, Additional file 6: Table S6, Additional file 7: Table S7 and Additional file 8: Table S8). Thirteen were diagnosed with CCD, 7 with MmD, 8 with non-specific histological features (classified as *RYR1*-related myopathy or RRM), and 1 with congenital muscular dystrophy. Onset ranged from birth to adulthood. The majority of patients with recessive disease were non-ambulatory (10/14), while most patients with dominant inheritance were ambulatory (11/15). Age at clinical review was not ascertained in the dataset and, therefore, may be a confounder for this result. Four of the patients (3 recessive, 1 dominant) required ventilatory support. Detailed clinical and pathologic information is included in Additional file 5: Table S5, Additional file 6: Table S6, Additional file 7: Table S7 and Additional file 8: Table S8.

There were 14 mutations found in the 14 dominant families (Additional file 7: Table S7). 7 (all missense) had been previously reported [8,35-39]. In terms of the novel variants, 4 are missense changes that are predicted (based on proximity to known mutations within the gene, on PolyPhen prediction, and on inheritance pattern) to be pathogenic/deleterious. One variant (patient N) is a splice site change that is presumed to be pathogenic because it segregates with disease in the family. The variant in patient T is predicted to alter splicing and result in an in-frame deletion of 25 amino acids. This variant is found in multiple affected family members.

There were 25 total sequence variants found in the 12 recessive families (Additional file 5: Table S5). 4 of the variants (1 nonsense and 3 missense) have been previously reported [12,31], while 21 were novel. 6 of the 21 novel mutations are predicted to result in the introduction of a premature stop codon. 13/15 of the remaining variants are missense changes, and there is one single amino acid deletion and one duplication. The missense changes were presumed to be pathogenic based on several factors, including clinical context, mutation inheritance, and PolyPhen prediction. Only the recessive mutations were included in the subsequent genotype-phenotype analyses.

### Recessive RYR1 mutation cohort: histopathologic subtypes and mutation association

**Figure 1:**
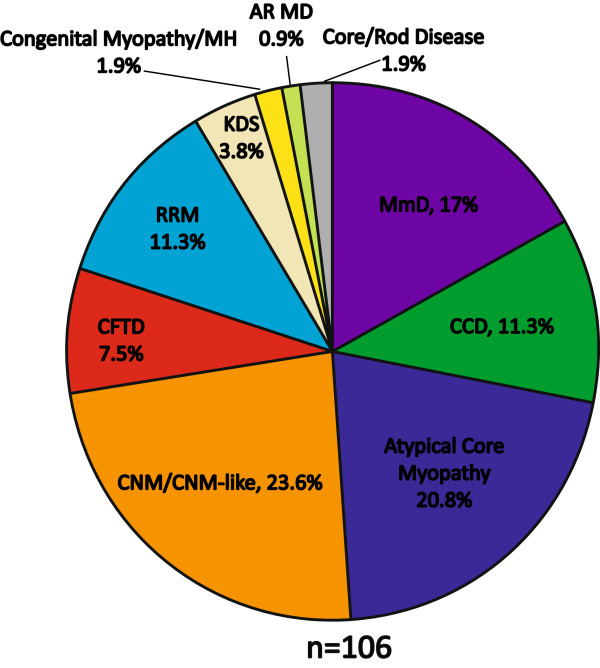
**Breakdown of hispathologic diagnosis.** Percentage breakdown of cases in the combined cohort by their predominant histopathologic pattern on muscle biopsy. Exceptions included are King Denborough Syndrome (KDS), which is a clinical diagnosis based primarily on specific dysmorphic features, and congenital myopathy plus malignant hyperthermia (MH), where individuals had non-specific biopsy features plus a history of MH. Abbreviations: multimincore disease (MmD), central core disease (CCD), centronuclear myopathy (CNM), congenital fiber type disproportion (CFTD), *RYR1*-related myopathy (RRM), autosomal recessive muscular dystrophy (AR MD).

A total of 106 cases with recessive *RYR1* mutations were further analyzed. These included the 14 new cases mentioned above and 92 cases identified in the medical literature (Additional file 1: Table S1, which includes the specific references for the previously published cases). The histopathologic diagnoses for all cases are summarized in Figure 1. The most highly represented were the core myopathies (51%) (inclusive of MmD, CCD, atypical-core myopathy, and core/rod disease), followed by CNM/CNM-like myopathies (23.6%).

**Figure 2:**
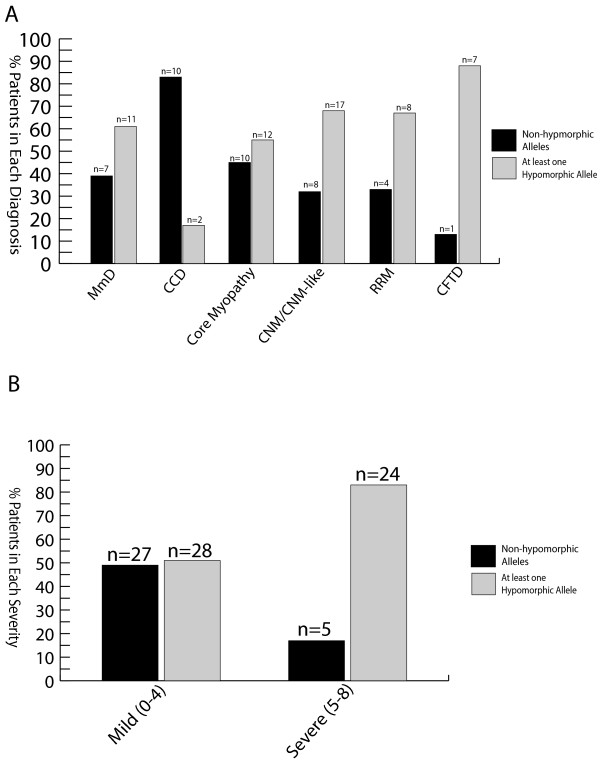
**Assessment of associations with mutation type. (A)** Mutation types among histopalogic diagnosis. Abbreviations: multimincore disease (MmD), central core disease (CCD), centronuclear myopathy (CNM), *RYR1*-related myopathy (RRM), congenital fiber type disproportion (CFTD). **(B)** Mutation types among severity groups. Clinical severity scores of 0–4 were considered mild, while scores of 5–8 were considered severe based on the criteria listed in Additional file 3: Table S3.

We first examined the relationship between mutation type and histopathologic diagnosis, and several patterns emerged from this analysis (Figure 2A). Among patients with MmD and atypical core myopathies, there were approximately equal percentages of patients with two non-hypomorphic (missense or in-frame indel) mutations and patients with at least one hypomorphic allele. On the other hand, the majority of patients with CCD had two non-hypomorphic mutations. The majority of patients with CNM/CNM-like, RRM and CFTD had at least one hypomorphic allele, although patient numbers were small for many of these subgroups.

### Mutation type, protein expression and clinical severity

Mutation type was next compared with clinical severity (Figure 2B) using a severity scale based on ambulation and respiratory status (Additional file 3: Table S3). A significant association was identified between mutation type and clinical severity: a larger proportion (83%) of patients with a severe phenotype (score ≥ 5 on 8 point scale) had at least one hypomorphic allele compared to patients with a mild phenotype (score of ≤ 4) (51%) (p = 0.0043). Another important factor on disease severity is the effect of the mutation on protein expression. There was a trend for low RyR1 protein levels (measured by western blot from patient biopsies) to be associated with a severe phenotype (Table 1), although data was available on relatively few biopsies (n = 14) and was not statistically significant (p = 0.14). Conversely, patients with only missense or small in-frame indel mutations (and no hypomorphic mutations) were significantly more likely to have a mild clinical phenotype. Of note, the likelihood of a severe phenotype was similar for all histological patterns. One bias to this observation is that age was not included in this analysis. Severity may be overestimated in patients too young to achieve certain motor milestones or may be overestimated in some patients as symptomology may improve with age.

**Table 1:** RYR1 protein level and clinical severity

**Patient identifier**	**RyR1 protein level (% of normal)**	**Severity rating**
**Mild 0-4**		
21	40+/−6	0
167	44	1
178	44+/−4	1
S	55	1
168	16	2
37	51+/−7	3
54	22+/−12	4
**Severe 5-8**		
179	14+/−3	5
55	15+/−8	6
123	10	6
132	38+/−4	7
109	58+/−3	8

RyR1 protein levels are presented as % of normal +/− standard error of the mean. Clinical severity scores of 0–4 were considered mild, while scores of 5–8 were considered severe based on the criteria listed in Additional file 3: Table S3.

### Mutation location, histopathologic subtype, and clinical severity

**Figure 3:**
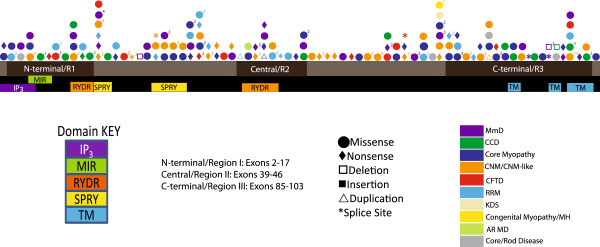
**Assessment of associations with mutation type.** Each mutation is represented by a shape which corresponds to the mutation type and color which corresponds to the diagnosis. Superscripts correspond to the number of times the mutation has been reported in separate families. Several regions of *RYR1* have been highlighted, including the mutation hotspots (Regions I-III) and domains. Abbreviations: multimincore disease (MmD), central core disease (CCD), centronuclear myopathy (CNM), congenital fiber type disproportion (CFTD), *RYR1*-related myopathy (RRM), King Denborough syndrome (KDS), malignant hyperthermia (MH), autosomal recessive muscular dystrophy (AR MD).

Non-hypomorphic alleles were present throughout the gene (Figure 3), and further analysis was needed to identify associations with mutation type, histopathologic subtype and clinical severity. In total, 102 different non-hypomorphic mutations were present in the 106 recessive *RYR1* patients we ascertained. We mapped the positions of these mutations to the known functional domains of RyR1. Analysis of the full cohort of non-hypomorphic mutations showed enrichment of mutations overall in MH/CCD hotspot regions (52% compared to an expected percentage based on RyR1 size of 37.8%, 99% confidence interval of 39-54%), though no significant enrichment in any one specific hotspot domain. We next examined more specifically whether there is an association between the position of mutations and either histological diagnosis (Table 2) or clinical severity (Table 3). Of note, by visual inspection there appears to be additional regions in *RYR1* where there are clusters of non-hypomorphic alleles. Because these clusters fell outside of any known functional region, we did not analyze them further.

**Table 2:** **Proportions of recessive non-hypomorphic missense mutations in known functional *RYR1 *domains analyzed by histological diagnosis**

		**Diagnostic categories**						
		**All mutations**^**+**^	**CCD**	**MmD**	**Atypical core**	**CNM/CNM-**	**CFTD**	**RYR1-related**
		**(N = 102)**	**(N = 17)**	**(N = 16)**	**myopathy**	**like myopathy**	**(N = 5)**	**myopathy**
					**(N = 27)**	**(N = 21)**		**(N = 10)**
**Domains**	**Expected %^**	**Observed % (CI)**	**Observed % (CI)**	**Observed % (CI)**	**Observed % (CI)**	**Observed % (CI)**	**Observed % (CI)**	**Observed % (CI)**
**MH/CCD hotspot domains**	37.8	52 (39–64) **	88 (53–98) **	38 (19–61)	52 (34–69)	43 (25–64)	0 (0–40)	60 (31–83)
Hotspot domain 1	11.5	16 (10–24)	24 (9–47)	13 (4–36)	11 (4–28)	5 (1–22)	0 (0–40)	30 (11–60)
Hotspot domain 2	5.9	10 (5–17)	6 (1–27)	13 (4–36)	11 (4–28)	5 (1–22)	0 (0–40)	30 (8–68)**
Hotspot domain 3	20.4	26 (19–36)	59 (30–83) **	13 (4–36)	30 (16–49)	33 (17–55)	0 (0–40)	0 (0–28)
**Selectivity filter**^**o**^	0.002%	3 (0.7- 11)**	18 (5–49)**	0 (0–20)	0 (0–13)	0 (0–16)	0 (0–43)	0 (0–28)
**Triadin**	1.2	6 (2–15) **	18 (5–49) **^a^	6 (1–28)	4 (1–18)	5 (1–22)	0 (0–43)	0 (0–28)
**DHPR**	31.4	**22 (15–31)**	**6 (0–27)**	19 (7–43)	19 (8–37)	19 (8–40)	20 (4–62)	60 (31–83)
**SPRY domains**	8.0	10 (5–17)	6 (1–27)	6 (1–28)	0 (0–13)	14 (5–35)	20 (4–62)	10 (2–40)
**S100A1**	12.0	5 (2–11)	6 (1–27)	6 (1–28)	4 (1–18)	0 (0–16)	20 (4–62)	10 (2–40)
**apoCaM**	11.0	7 (3–14)	0 (0–18)	0 (0–20)	15 (6–33)	14 (4–35)	0 (0–43)	0 (0–28)
**CaCaM**	2.7	1 (0–5)	0 (0–18)	0 (0–20)	4 (1–18)	0 (0–16)	0 (0–43)	0 (0–28)
**Interdomain interactions**	1.5	1 (0–5)	0 (0–18)	0 (0–20)	0 (0–13)	5 (1–22)	0 (0–43)	0 (0–28)

^+^ Six mutations are only included in the ‘All mutations’ analysis as histological information was not available for further classification. ^ Expected percentage (%) = the percentage of mutations in each domain that would occur by chance alone, based on the size of the domain relative to the full RyR1 amino acid sequence. Observed % = percentage of mutations in each functional domain. All Wilson confidence intervals (CI) denote 95% intervals unless indicated by **, which are 99% confidence intervals. Statistically significant results are shown in bold font. N = number of mutations in each diagnostic category. ^o^ Amino acids 4891–4900. ^a^ All CCD mutations in the triadin-binding region also lie in the selectivity filter of the pore (amino acids 4895–4900) and so are likely to have their main effects on ion conductivity directly, rather than on triadin binding. CCD = central core disease. MmD = multi-minicore disease. CFTD = congenital fiber type disproportion. MH = malignant hyperthermia. DHPR = dihydropyridine receptor. apoCaM = calmodulin without Ca^2+^ bound. CaCaM = calmodulin with Ca^2+^ bound.

**Table 3:** **An analysis of the position of all recessive non-hypomorphic *RYR1 *mutations vs severity** &**ophthalmoparesis**

	**Mutations associated with mild phenotypes/total cohort**	**Percentage of mutations with mild phenotypes (CI)**	**Mutations associated with ophthalmoplegia/**	**Percentage of mutations associated with ophthalmoplegia (CI)**
			**Total mutations**	
Total cohort*	46/67	68.7	43/102	42.2
**Domain**				
**Combined MH/CCD hotspot domains**	21/35	60 (44–74)	17/52	33 (21–46)
*Hotspot domain 1*	9/12	75 (46–92)	6/16	38 (18–61)
*Hotspot domain 2*	4/4	100 (54–100)	2/10	20 (5–52)
*Hotspot domain 3*	8/19	**42 (23–64)**	9/26	35 (19–54)
**Interdomain interactions**	1/1	100 (22–100)	1/1	100 (22–100)
**Triadin**	0/3	**0 (0–53)**	2/7	29 (8–65)
**DHPR**	9/10	90 (46–100)	5/20	25 (11–47)
**S100A1**	2/2	100 (22–100)	1/5	20 (2–64)
**apoCaM**	4/4	100 (37–100)	4/7	57 (25–84)
**CaCaM**	0/0	-	0/1	0 (0–78)
**SPRY domains**	3/5	60 (17–92)	7/11	64 (35–85)

Severity scores between 0 and 4 were classed as mild and scores between 5 and 8 were classed as severe (Additional file 3: Table S3). Two results in the analysis of disease severity vs mutation domain deviated significantly from expected ratios. Mutations in MH/CCD hotspot 3 and in the triadin binding domain (with sits within hotspot 3) were associated with a severe phenotype more often than expected by chance. There was no evidence that non-hypomorphic mutations in specific domains were associated with a greater or lower chance of ophthalmoparesis. * To reduced bias, each non-hypomorphic recessive RYR1 mutation was counted only once, even if several individuals have been reported with the mutation. For the analysis of phenotype severity, severity scores were averaged when severity data was available on several individuals with a mutation. For the analysis of ophthalmoparesis, mutations were classified as associated with ophthalmoplaresis if any patient with the mutation was reported to have ophthalmoparesis. CI = 95% Wilson confidence intervals. Bold font shows where results are significantly different from expected ratios.

Subgroup analysis for histological diagnosis showed an association most strongly for CCD, particularly in MH/CCD hotspot region 3 (Table 2), a C-terminal region that spans residues 3916–4942. 59% of non-hypomorphic CCD mutations were located in hotspot region 3, a significant increase (99% confidence interval between 30-83%) compared to an expected percentage (based on its size compared to the overall size of RyR1) of only 20.4%. The selectivity filter of the channel pore (Gly4891-D4900) which sits within the triadin-binding region and MH/CCD hotspot 3, was also significantly enriched for mutations in CCD patients (18% of mutations compared to 0.002% expected by size, with 99% confidence interval of 5-49%).

Subgroup analysis identified other possible associations with histopathologic diagnosis as well. The DHPR-binding domains had fewer mutations than expected in the full mutation cohort and CCD subgroup analysis (Table 2). Mutations associated with non-specific histological patterns clustered more than expected in MH/CCD hotspot regions 2. However, no associations with mutation position were seen for MmD, CNM or CFTD.

In terms of clinical severity, two observations emerged from the analysis of non-hypomorphic mutations. These were that mutations in MH/CCD hotspot 3 (58% association, n = 19 mutations) and in the triadin binding domain (100% association, though for n = 3), which sits within MH/CCD hotspot 3, were associated with a severe phenotype more often than expected by chance and than with the overall cohort (31.3%) (Table 3). Of note, we only were able to derive severity scores for 67 of the non-hypomorphic mutations, which thus limited the scope of this particular analysis.

### Mutation type/location and ophthalmoparesis

**Figure 4:**
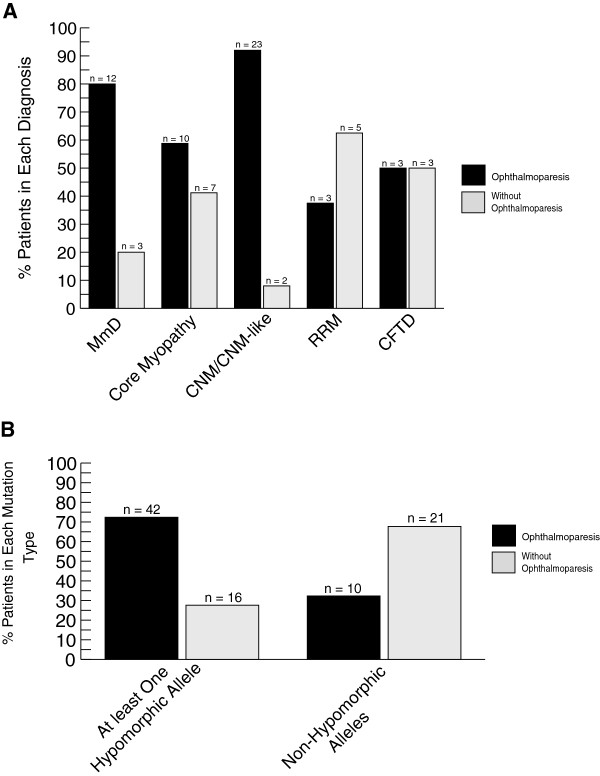
**Assessment of associations with ophthalmoparesis. (A)** Ophthalmoparesis among hispathologic diagnosis. Abbreviations: multimincore disease (MmD), centronuclear myopathy (CNM), *RYR1*-related myopathy (RRM), congenital fiber type disproportion (CFTD). **(B)** Ophthalmoparesis among mutation types.

In terms of specific clinical symptoms, ophthalmoparesis is a key clinical feature found in a subset of patients with *RYR1* mutations. In some contexts (e.g. minicore myopathy) it can be used to help distinguish patients with *RYR1* mutations from other genetic subtypes [40]. We examined for an association between ophthalmoparesis and histopathological subtype, mutation type and clinical severity. Several associations were identified (Figure 4). Among all histopathological subtypes, ophthalmoparesis was more likely to be associated with a diagnosis of CNM/CNM-like (p = 0.006). Additionally, a larger proportion of patients with at least one hypomorphic allele (72%) had ophthalmoparesis than those with non-hypomorphic mutations (32%) (p = 0.0003). Conversely, there was no significant association between ophthalmoparesis and clinical severity (data not shown) or between ophthalmoparesis and mutation position (Table 3).

## Discussion

There has been a recent explosion in the identification of new cases of congenital myopathies due to *RYR1* mutations, particularly those with recessive inheritance. It is thus now possible to perform mutation correlations that are adequately powered to uncover significant associations. In this study, we present a comprehensive genotype-phenotype analysis of *RYR1* mutations from more than 100 cases of recessively inherited *RYR1*-related myopathies. This study represents the largest examination of recessive cases to date, and includes 14 new cases plus analysis of all recessive cases in the medical literature that we could ascertain. Using this cohort, we have identified several statistically significant associations that provide clues about disease mechanisms in different histological diagnoses and about important determinants of disease severity. The most notable finding is an association between increased disease severity and the presence of at least one hypomorphic (nonsense, frame shift, splice site) allele. Additional important observations include the identification of enrichment of missense mutations in specific locations associated with particular histopathologic subtypes of *RYR1*-related myopathy, suggesting that histological phenotype is at least partially determined by mutation position.

### General cohort statistics: core vs non-core myopathies

Historically, the overwhelming majority of congenital myopathy patients with mutations in *RYR1* had a core myopathy. The discovery of *RYR1* mutations in cases of non-core myopathy is a relatively recent phenomenon, and the relative prevalence of such cases has been uncertain. We were surprised to find that 49% of recessive cases identified in our study were non-core myopathies. Once ascertainment bias is removed (i.e. a historical skewing toward *RYR1* mutation identification in core myopathies because these were the first *RYR1* myopathies identified), it is possible that non-core myopathies ultimately account for more than 50% of recessive *RYR1* disease. However, a potential caveat to this assertion is a reverse bias created by the fact that some patients with core myopathy (particularly central core disease) are assumed to have *RYR1* mutations and thus not confirmed at the genetic level.

### Mutation type and clinical severity: hypomorphic alleles are associated with severe disease

One of our primary goals was to see whether disease severity could be predicted based on the type of mutation. We found a clear, statistically significant association between the presence of a hypomorphic mutation and a severe clinical picture. This data is in keeping with analyses of smaller patient cohorts, and suggests that reduced total RyR1 protein levels is an important disease mechanism that heralds more severe disease [22]. Of note, no patient has yet been reported with two fully hypomorphic *RYR1* mutations, suggesting that some residual RyR1 function is required for life. This is in keeping with the perinatal lethal phenotype of *Ryr1* knockout mice [41]. An extrapolation of this hypothesis is that all recessive missense/indel mutations that are found in association with a hypomorphic mutation or that are present in a homozygous state are unlikely to completely abolish RyR1 function. One can also predict that increasing expression of RyR1, even of RyR1 subunits with abnormal characteristics due to missense or other mutations, may serve to ameliorate disease. Additional research will be necessary to test this hypothesis.

### Mutations, diagnosis, and functional domains

We also sought to identify patterns between mutation type/location and specific histopathologic diagnoses as they may give insights into the pathogenesis of the different histological patterns that can arise. One significant trend identified was that hypomorphic mutations were more likely to be seen in non-CCD myopathy subtypes compared to central core disease. This suggests that non-central core pathology is, in part, dictated by reduction in RyR1 expression and presumably reduced RyR1 function. This would be consistent with animal model data, particularly from the zebrafish model of *RYR1*-myopathy, which has severely reduced expression (>90% reduction), obvious myopathic features, but no central cores and very few true minicores [7,42]. One explanation may be that CCD is often due (particularly in de novo/dominant causes) to mutations that result in the expression of RyR1 channels with abnormal properties while all other histological phenotypes are associated with a generalized reduction in RyR1 function.

We also identified several interesting associations between clinical/histological phenotypes and mutation location. Previous studies have found no patterns in the location of recessive mutations, and a prevailing assumption has been that recessive mutations are randomly distributed throughout the gene [1]. However, our analysis reveals that there are significant enrichments of mutations in known functional domains of the protein and in association with particular diagnoses. In particular, we detected an over-representation of recessive non-hypomorphic *RYR1* mutations in the combined MH/CCD hotspots and in the subgroup analysis there was a strong association between CCD mutations and hotspot region 3 that exceeded 99% confidence intervals. This is the same pattern that is seen for dominant CCD mutations, which are more often in hotspot region 3 than regions 1 or 2 [8] and suggests that dominant and recessive forms of CCD may share similar disease mechanisms.

One association that provides a clue about disease pathogenesis is the enrichment of recessive missense mutations in the selectivity filter of the channel pore, a 10 amino acid motif that conserved in all RyRs and that is critical for channel function [43]. This region only occupies 0.002% of the linear protein sequence, but accounts for 3% of recessive non-hypomorphic mutations overall and almost 20% of recessive CCD mutations. This implicates abnormalities in ion conductance or ion selectivity in the pathogenesis of recessive CCD, a mechanism that has been linked to dominant CCD mutations (reviewed in [9,27]). The enrichment of recessive missense mutations in the triadin-binding domain is due to the fact that this domain contains the selectivity filter. The finding that fewer mutations than expected were located in DHPR binding regions may indicate that interactions with these RyR1-binding proteins tend not to be important in the pathogenesis of the known *RYR1* myopathies. Some associations are expected by chance when making multiple comparisons and it would be ideal to replicate these findings in a second cohort of mutations as confirmation.

## Conclusions

In all, we present a comprehensive genotype-phenotype study of recessive *RYR1* mutations. The insights we provide reveal the utility of such a study, and show the first associations between mutation type and location and clinical severity and diagnosis. In particular, we highlight the MH/CCD hotpspot regions, (and the selectivity filter that lies within hotspot region 3), as likely to be important in the pathogenesis of many recessive *RYR1*-related myopathies. The study of additional patients in the future will confirm and refine these associations.

## Competing interests

The authors declare that they have no competing interests. MT was paid consulting fees by Biomarin in relation to an unrelated clinical trial, and was paid by Genzyme/Sanofi for lecture and travel costs associated with the Steps Forward Meeting (2009, 2011, 2012).

## Authors’ contributions

KA participated in the design and coordination of the study, assisted in data collection and interpretation, and drafted the manuscript. AB was responsible for data collection and interpretation. JH assisted with data management and helped to draft the manuscript. MT, CB, LM, DM, JC, JRD, and GW provided new cases of RYR1 myopathy and also provided critical reading of the manuscript. BC performed the statistical analysis. JJD and NC conceived the study, participated in data analysis, and drafted and edited the manuscript. All authors read and approved the final manuscript.

## Supplementary Material

Additional file 1: Table S1 Literature review of recessive *RYR1* mutations. References for all cases in the literature are included at the end of the spreadsheet and in the manuscript. No diagnosis refers to those cases where histologic information as not available to further define the diagnosis. Click here for file

Additional file 2: Table S2 Grouping patients into broad diagnostic categories for analysis. Click here for file

Additional file 3: Table S3 Criteria for severity scores. Ambulatory and respiratory ratings were added together to calculate the overall severity score. Abbreviations: pulmonary function testing (PFT), ventilator (Vent), continuous positive airway pressure (CPAP), bilevel positive airway pressure (BiPAP). Click here for file

Additional file 4: Table S4 Functional domains in the ryanodine receptor type1. *Amino acids are numbered relative to the full RyR1 amino acid sequence (NM_000540). DHPR = dihydropyridine receptor, apoCaM = calmodulin without bound Ca2+, CaCaM = calmodulin with bound Ca2+. Information summarized from Hwang et al., 2012. Click here for file

Additional file 5: Table S5 Clinical characteristics of newly reported recessive *RYR1* mutations. Severity scores based on criteria listed in Additional file 3: Table S3. Also included are the PolyPhen2 predictions for all novel missense mutations (Probably Damaging (PRD), Possibly Damaging (POD)) and the severity (scale 0–1, 0 indicating least severe, 1 indicating most severe). Origin of the mutation is designated ^M^ for maternal and ^P^ for paternal. For patients F&G, carrier testing was only performed on the father. Since one mutation was identified, the other mutation is presumed to be maternal, which is denoted by ^M*^. Patients O and P (siblings) were presumed recessive based on clinical presentation, lack of parental symptoms, and the presence of two mutations. Parental testing was not performed, so it is formally possible (though unlikely) that the two mutations exist in cis. For patient Y, one mutation was identified as maternal in origin. Carrier testing of the father did not identify the second mutation, therefore, it is presumed to be *de novo*. Abbreviations: Patient ID (ID), siblings (F&G, O&P) (*), diagnosis (DX), multimincore disease (MmD), core myopathy (CM), *RYR1*-related myopathy (RRM), autosomal recessive muscular dystrophy (AR MD), central core disease (CCD), first year of life (FYOL), Weakness: proximal (P), distal (D), facial (F), neck (N); rigid spine (RS), ophthalmoparesis (OPH), respiratory distress (RD), ventilator (Vent), feeding difficulties (FD), malignant hyperthermia (MH), creatine kinase (CK). Previously reported mutations: ^a^Bevilacqua, et al., 2011, ^b^Zhou, et al., 2010. Click here for file

Additional file 6: Table S6 Hispathologic findings of newly reported recessive *RYR1* mutations. Diagnosis of cases without a muscle biopsy was based on family history of the disease. Mutations previously reported in the medical literature (^a^Bevilacqua, et al., 2011, ^b^Zhou, et al., 2010). Origin of the mutation is designated ^M^ for maternal and ^P^ for paternal. For patients F&G, carrier testing was only performed on the father. Since one mutation was identified, the other mutation is presumed to be maternal, which is denoted by ^M*^. For patient Y, one mutation was identified as maternal in origin. Carrier testing of the father did not identify the second mutation, therefore, it is *de novo* which is denoted by ^D^. Abbreviations: Patient ID (ID), siblings (F&G, O&P) (*), diagnosis (DX), multimincore disease (MmD), core myopathy (CM), *RYR1*-related myopathy (RRM), autosomal recessive muscular dystrophy (AR MD), central core disease (CCD), central cores (CC), minicores (MC), internalized nuclei (IN), central nuclei (CN). Click here for file

Additional file 7: Table S7 Clinical characteristics of newly reported dominant *RYR1* mutations. Severity scores based on criteria listed in Additional file 3: Table S3. Polyphen2 scores are also included for all novel missense mutations (Probably Damaging (PRD)) and the severity (scale 0–1, 0 indicating least severe, 1 indicating most severe). ^ indicates that the silent mutation is predicted to create a new splice donor site resulting in a 25 amino acid in-frame deletion. Previously reported mutations: ^a^Lynch, et al., 1999, ^b^Davis, et al., 2003, ^c^Manning, et al., 1998, ^d^Chamley, et al., 2000, ^e^Monnier, et al., 2001, ^f^Davis, et al., 2002). Origin of the mutation is designated ^M^ for maternal, ^P^ for paternal, or ^D^ for *de novo*. Abbreviations: Patient ID (ID), siblings (B&C), diagnosis (DX), central core disease (CCD), *RYR1*-related myopathy (RRM), multimincore disease (MmD), first year of life (FYOL), Weakness: proximal (P), distal (D), facial (F), neck (N); rigid spine (RS), ophthalmoparesis (OPH), respiratory distress (RD), ventilator (Vent), feeding difficulties (FD), malignant hyperthermia (MH), creatine kinase (CK). Click here for file

Additional file 8: Table S8 Hispathologic findings of newly reported dominant *RYR1* mutations. Previously reported mutations: ^a^Lynch, et al., 1999, ^b^Davis, et al., 2003, ^c^Manning, et al., 1998, ^d^Chamley, et al., 2000, ^e^Monnier, et al., 2001, ^f^Davis, et al., 2002) [8,36-39,44]. Origin of the mutation is designated ^M^ for maternal, ^P^ for paternal, or ^D^ for *de novo*. Abbreviations: Patient ID (ID), siblings (B&C), diagnosis (DX), central core disease (CCD), *RYR1*-related myopathy (RRM), multimincore disease (MmD), central cores (CC), minicores (MC), internalized nuclei (IN), central nuclei (CN). Click here for file
