# Supplementary material for: Genotype-phenotype correlations in recessive RYR1-related myopathies
Source: Orphanet J Rare Dis. 2013 Aug 6;8:117. doi: 10.1186/1750-1172-8-117 (PMC3751094; doi:10.1186/1750-1172-8-117)
Supplement: Additional file 2: Table S2 — Grouping patients into broad diagnostic categories for analysis. [file 1750-1172-8-117-S2.docx]

| **Diagnostic category** | **Reported by pathologists or by authors of case reports in the literature** |
| --- | --- |
| Central core disease (CCD) | Central core disease; prominent central cores |
| Multi-minicore disease (MmD) | Multi-minicore disease; prominent minicores |
| Atypical core myopathy | Atypical core myopathy; core myopathy; core-rod myopathy; Notable cores or, irregular or patchy staining on oxidative stains. |
| CNM/CNM-like myopathy | Centronuclear myopathy; CNM-like myopathy; Markedly increased internalised nuclei present as the most notable histological abnormality |
| CFTD | Congenital fibre type disproportion |
| RYR1-related myopathy (RRM) | Histological abnormalities were not consistent with another category |
