# Supplementary material for: Genotype-phenotype correlations in recessive RYR1-related myopathies
Source: Orphanet J Rare Dis. 2013 Aug 6;8:117. doi: 10.1186/1750-1172-8-117 (PMC3751094; doi:10.1186/1750-1172-8-117)
Supplement: Additional file 3: Table S3 — Criteria for severity scores. Ambulatory and respiratory ratings were added together to calculate the overall severity score. Abbreviations: pulmonary function testing (PFT), ventilator (Vent), continuous positive airway pressure (CPAP), bilevel positive airway pressure (BiPAP). [file 1750-1172-8-117-S3.pptx]

## Slide 1
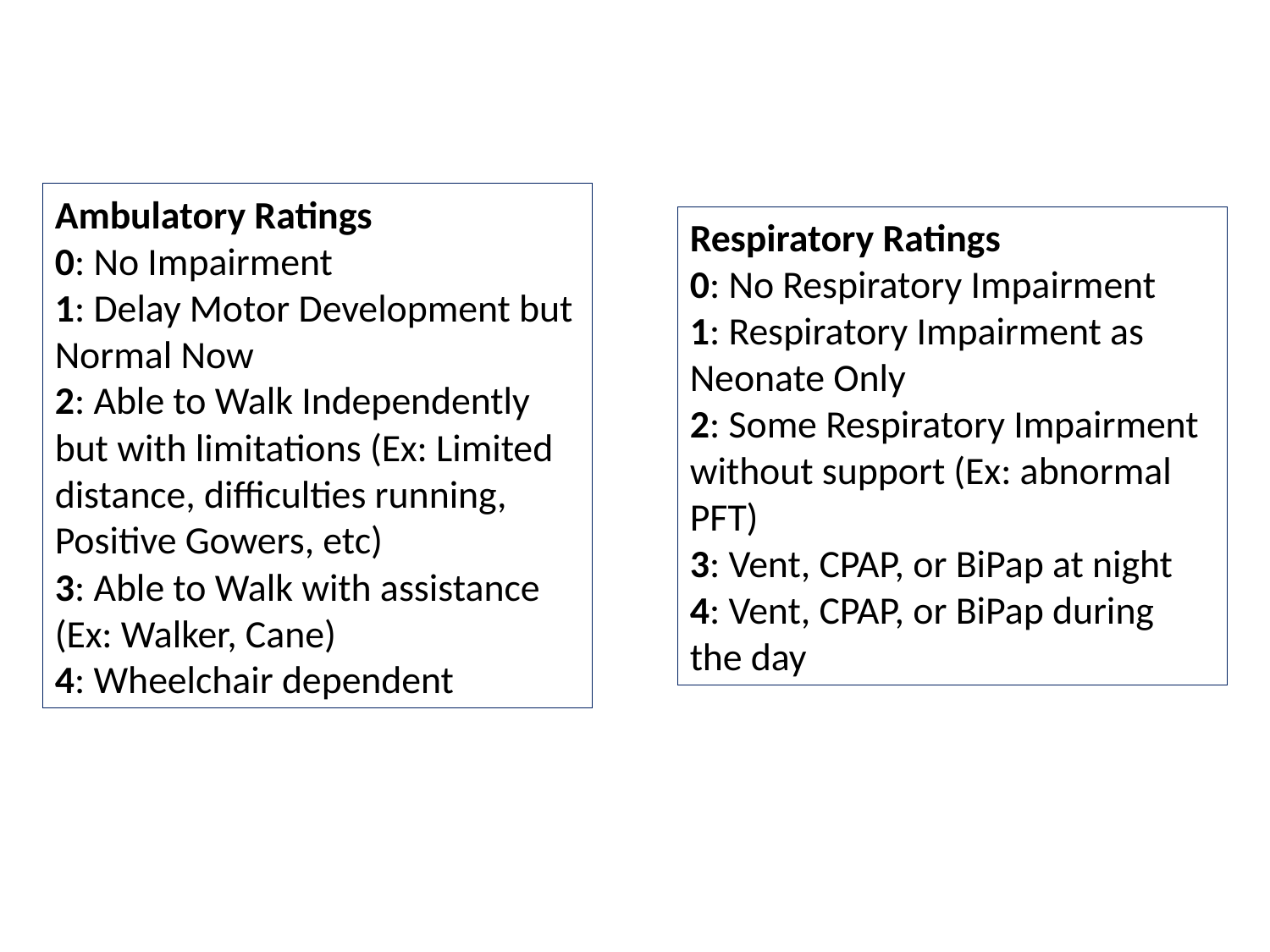

Ambulatory Ratings
0: No Impairment
1: Delay Motor Development but Normal Now
2: Able to Walk Independently but with limitations (Ex: Limited distance, difficulties running, Positive Gowers, etc)
3: Able to Walk with assistance (Ex: Walker, Cane)
4: Wheelchair dependent
Respiratory Ratings
0: No Respiratory Impairment
1: Respiratory Impairment as Neonate Only
2: Some Respiratory Impairment without support (Ex: abnormal PFT)
3: Vent, CPAP, or BiPap at night
4: Vent, CPAP, or BiPap during the day
