# Supplementary material for: Genotype-phenotype correlations in recessive RYR1-related myopathies
Source: Orphanet J Rare Dis. 2013 Aug 6;8:117. doi: 10.1186/1750-1172-8-117 (PMC3751094; doi:10.1186/1750-1172-8-117)
Supplement: Additional file 4: Table S4 — Functional domains in the ryanodine receptor type1. *Amino acids are numbered relative to the full RyR1 amino acid sequence (NM_000540). DHPR = dihydropyridine receptor, apoCaM = calmodulin without bound Ca2+, CaCaM = calmodulin with bound Ca2+. Information summarized from Hwang et al., 2012. [file 1750-1172-8-117-S4.docx]

| **Domain** | **Amino acids in domain*** | **Proportion of total RyR1 protein** |
| --- | --- | --- |
| **Combined MH/CCD Hotspot Domains** | 35-614, 2163-2458, 3916-4942 | 37.8% |
| *Hotspot Domain 1* | 35-614 | 11.5% |
| *Hotspot Domain 2* | 2163-2458 | 5.9% |
| *Hotspot Domain 3* | 3916-4942 | 20.4% |
| **Interdomain Interactions** | 589-608, 2442-2477, 4821-4842 | 1.5% |
| **Triadin** | 4861-4918 | 1.2% |
| **DHPR** | 1085-1208, 1341-1402, 1635-2636, 3495-3499 | 23.7% |
| **S100A1** | 1861-2155, 3616-3627, 3773-3873, 4426-4622 | 12% |
| **apoCaM** | 1975-1999, 2937-3225, 3546-3655, 4303-4431 | 11% |
| **CaCaM** | 1975-1999, 3553-3662 | 2.7% |
| **SPRY Domains** | 659-797, 1085-1208, 1430-1570 | 8% |
