# Supplementary material for: Genotype-phenotype correlations in recessive RYR1-related myopathies
Source: Orphanet J Rare Dis. 2013 Aug 6;8:117. doi: 10.1186/1750-1172-8-117 (PMC3751094; doi:10.1186/1750-1172-8-117)
Supplement: Additional file 5: Table S5 — Clinical characteristics of newly reported recessive RYR1 mutations. Severity scores based on criteria listed in Additional file 3: Table S3. Also included are the PolyPhen2 predictions for all novel missense mutations (Probably Damaging (PRD), Possibly Damaging (POD)) and the severity (scale 0–1, 0 indicating least severe, 1 indicating most severe). Origin of the mutation is designated M for maternal and P for paternal. For patients F&G, carrier testing was only performed on the father. Since one mutation was identified, the other mutation is presumed to be maternal, which is denoted by M*. Patients O and P (siblings) were presumed recessive based on clinical presentation, lack of parental symptoms, and the presence of two mutations. Parental testing was not performed, so it is formally possible (though unlikely) that the two mutations exist in cis. For patient Y, one mutation was identified as maternal in origin. Carrier testing of the father did not identify the second mutation, therefore, it is presumed to be de novo. Abbreviations: Patient ID (ID), siblings (F&G, O&P) (*), diagnosis (DX), multimincore disease (MmD), core myopathy (CM), RYR1-related myopathy (RRM), autosomal recessive muscular dystrophy (AR MD), central core disease (CCD), first year of life (FYOL), Weakness: proximal (P), distal (D), facial (F), neck (N); rigid spine (RS), ophthalmoparesis (OPH), respiratory distress (RD), ventilator (Vent), feeding difficulties (FD), malignant hyperthermia (MH), creatine kinase (CK). Previously reported mutations: aBevilacqua, et al., 2011, bZhou, et al., 2010. [file 1750-1172-8-117-S5.pptx]

## Slide 1
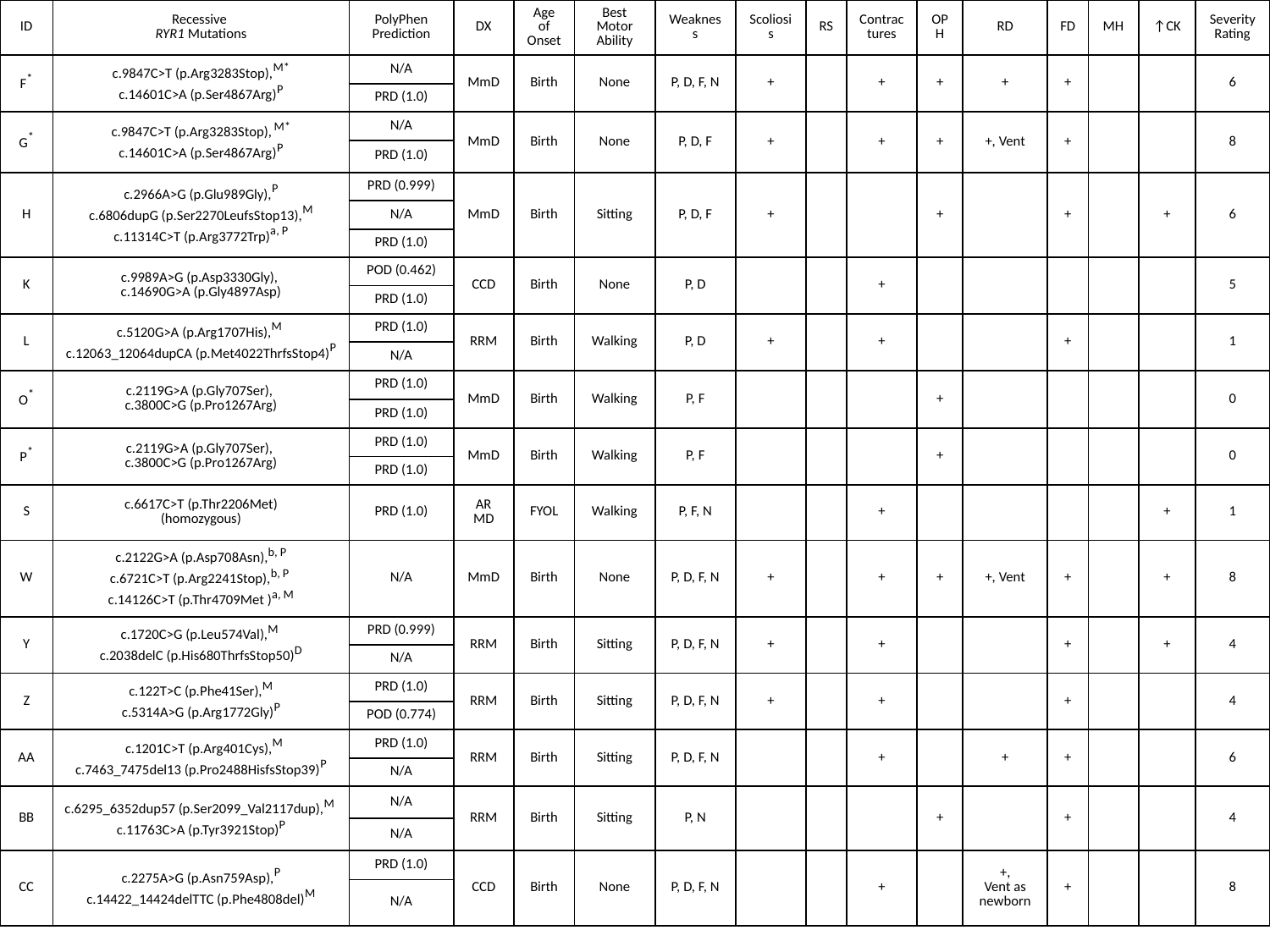

| ID | Recessive RYR1 Mutations | PolyPhen Prediction | DX | Age of Onset | Best Motor Ability | Weakness | Scoliosis | RS | Contractures | OPH | RD | FD | MH | ↑CK | Severity Rating |
| --- | --- | --- | --- | --- | --- | --- | --- | --- | --- | --- | --- | --- | --- | --- | --- |
| F\* | c.9847C>T (p.Arg3283Stop),M\* c.14601C>A (p.Ser4867Arg)P | N/A | MmD | Birth | None | P, D, F, N | + | | + | + | + | + | | | 6 |
| | | PRD (1.0) | | | | | | | | | | | | | |
| G\* | c.9847C>T (p.Arg3283Stop), M\* c.14601C>A (p.Ser4867Arg)P | N/A | MmD | Birth | None | P, D, F | + | | + | + | +, Vent | + | | | 8 |
| | | PRD (1.0) | | | | | | | | | | | | | |
| H | c.2966A>G (p.Glu989Gly),P c.6806dupG (p.Ser2270LeufsStop13),M c.11314C>T (p.Arg3772Trp)a, P | PRD (0.999) | MmD | Birth | Sitting | P, D, F | + | | | + | | + | | + | 6 |
| | | N/A | | | | | | | | | | | | | |
| | | PRD (1.0) | | | | | | | | | | | | | |
| K | c.9989A>G (p.Asp3330Gly), c.14690G>A (p.Gly4897Asp) | POD (0.462) | CCD | Birth | None | P, D | | | + | | | | | | 5 |
| | | PRD (1.0) | | | | | | | | | | | | | |
| L | c.5120G>A (p.Arg1707His),M c.12063\_12064dupCA (p.Met4022ThrfsStop4)P | PRD (1.0) | RRM | Birth | Walking | P, D | + | | + | | | + | | | 1 |
| | | N/A | | | | | | | | | | | | | |
| O\* | c.2119G>A (p.Gly707Ser), c.3800C>G (p.Pro1267Arg) | PRD (1.0) | MmD | Birth | Walking | P, F | | | | + | | | | | 0 |
| | | PRD (1.0) | | | | | | | | | | | | | |
| P\* | c.2119G>A (p.Gly707Ser), c.3800C>G (p.Pro1267Arg) | PRD (1.0) | MmD | Birth | Walking | P, F | | | | + | | | | | 0 |
| | | PRD (1.0) | | | | | | | | | | | | | |
| S | c.6617C>T (p.Thr2206Met) (homozygous) | PRD (1.0) | AR MD | FYOL | Walking | P, F, N | | | + | | | | | + | 1 |
| W | c.2122G>A (p.Asp708Asn),b, P c.6721C>T (p.Arg2241Stop),b, P c.14126C>T (p.Thr4709Met )a, M | N/A | MmD | Birth | None | P, D, F, N | + | | + | + | +, Vent | + | | + | 8 |
| Y | c.1720C>G (p.Leu574Val),M c.2038delC (p.His680ThrfsStop50)D | PRD (0.999) | RRM | Birth | Sitting | P, D, F, N | + | | + | | | + | | + | 4 |
| | | N/A | | | | | | | | | | | | | |
| Z | c.122T>C (p.Phe41Ser),M c.5314A>G (p.Arg1772Gly)P | PRD (1.0) | RRM | Birth | Sitting | P, D, F, N | + | | + | | | + | | | 4 |
| | | POD (0.774) | | | | | | | | | | | | | |
| AA | c.1201C>T (p.Arg401Cys),M c.7463\_7475del13 (p.Pro2488HisfsStop39)P | PRD (1.0) | RRM | Birth | Sitting | P, D, F, N | | | + | | + | + | | | 6 |
| | | N/A | | | | | | | | | | | | | |
| BB | c.6295\_6352dup57 (p.Ser2099\_Val2117dup),M c.11763C>A (p.Tyr3921Stop)P | N/A | RRM | Birth | Sitting | P, N | | | | + | | + | | | 4 |
| | | N/A | | | | | | | | | | | | | |
| CC | c.2275A>G (p.Asn759Asp),P c.14422\_14424delTTC (p.Phe4808del)M | PRD (1.0) | CCD | Birth | None | P, D, F, N | | | + | | +, Vent as newborn | + | | | 8 |
| | | N/A | | | | | | | | | | | | | |
