# Supplementary material for: Genotype-phenotype correlations in recessive RYR1-related myopathies
Source: Orphanet J Rare Dis. 2013 Aug 6;8:117. doi: 10.1186/1750-1172-8-117 (PMC3751094; doi:10.1186/1750-1172-8-117)
Supplement: Additional file 6: Table S6 — Hispathologic findings of newly reported recessive RYR1 mutations. Diagnosis of cases without a muscle biopsy was based on family history of the disease. Mutations previously reported in the medical literature (aBevilacqua, et al., 2011, bZhou, et al., 2010). Origin of the mutation is designated M for maternal and P for paternal. For patients F&G, carrier testing was only performed on the father. Since one mutation was identified, the other mutation is presumed to be maternal, which is denoted by M*. For patient Y, one mutation was identified as maternal in origin. Carrier testing of the father did not identify the second mutation, therefore, it is de novo which is denoted by D. Abbreviations: Patient ID (ID), siblings (F&G, O&P) (*), diagnosis (DX), multimincore disease (MmD), core myopathy (CM), RYR1-related myopathy (RRM), autosomal recessive muscular dystrophy (AR MD), central core disease (CCD), central cores (CC), minicores (MC), internalized nuclei (IN), central nuclei (CN). [file 1750-1172-8-117-S6.pptx]

## Slide 1
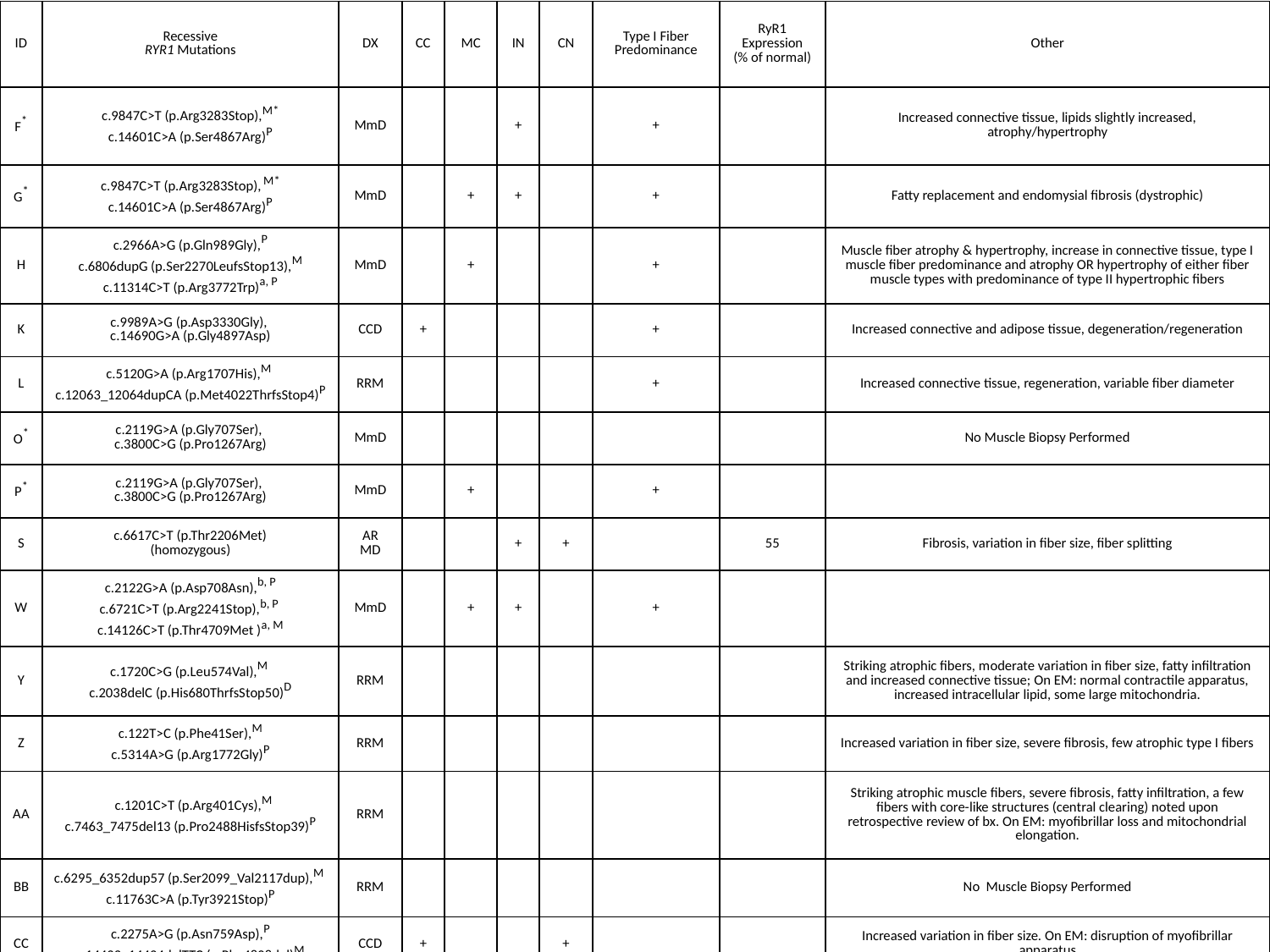

| ID | Recessive RYR1 Mutations | DX | CC | MC | IN | CN | Type I Fiber Predominance | RyR1 Expression (% of normal) | Other |
| --- | --- | --- | --- | --- | --- | --- | --- | --- | --- |
| F\* | c.9847C>T (p.Arg3283Stop),M\* c.14601C>A (p.Ser4867Arg)P | MmD | | | + | | + | | Increased connective tissue, lipids slightly increased, atrophy/hypertrophy |
| G\* | c.9847C>T (p.Arg3283Stop), M\* c.14601C>A (p.Ser4867Arg)P | MmD | | + | + | | + | | Fatty replacement and endomysial fibrosis (dystrophic) |
| H | c.2966A>G (p.Gln989Gly),P c.6806dupG (p.Ser2270LeufsStop13),M c.11314C>T (p.Arg3772Trp)a, P | MmD | | + | | | + | | Muscle fiber atrophy & hypertrophy, increase in connective tissue, type I muscle fiber predominance and atrophy OR hypertrophy of either fiber muscle types with predominance of type II hypertrophic fibers |
| K | c.9989A>G (p.Asp3330Gly), c.14690G>A (p.Gly4897Asp) | CCD | + | | | | + | | Increased connective and adipose tissue, degeneration/regeneration |
| L | c.5120G>A (p.Arg1707His),M c.12063\_12064dupCA (p.Met4022ThrfsStop4)P | RRM | | | | | + | | Increased connective tissue, regeneration, variable fiber diameter |
| O\* | c.2119G>A (p.Gly707Ser), c.3800C>G (p.Pro1267Arg) | MmD | | | | | | | No Muscle Biopsy Performed |
| P\* | c.2119G>A (p.Gly707Ser), c.3800C>G (p.Pro1267Arg) | MmD | | + | | | + | | |
| S | c.6617C>T (p.Thr2206Met) (homozygous) | AR MD | | | + | + | | 55 | Fibrosis, variation in fiber size, fiber splitting |
| W | c.2122G>A (p.Asp708Asn),b, P c.6721C>T (p.Arg2241Stop),b, P c.14126C>T (p.Thr4709Met )a, M | MmD | | + | + | | + | | |
| Y | c.1720C>G (p.Leu574Val),M c.2038delC (p.His680ThrfsStop50)D | RRM | | | | | | | Striking atrophic fibers, moderate variation in fiber size, fatty infiltration and increased connective tissue; On EM: normal contractile apparatus, increased intracellular lipid, some large mitochondria. |
| Z | c.122T>C (p.Phe41Ser),M c.5314A>G (p.Arg1772Gly)P | RRM | | | | | | | Increased variation in fiber size, severe fibrosis, few atrophic type I fibers |
| AA | c.1201C>T (p.Arg401Cys),M c.7463\_7475del13 (p.Pro2488HisfsStop39)P | RRM | | | | | | | Striking atrophic muscle fibers, severe fibrosis, fatty infiltration, a few fibers with core-like structures (central clearing) noted upon retrospective review of bx. On EM: myofibrillar loss and mitochondrial elongation. |
| BB | c.6295\_6352dup57 (p.Ser2099\_Val2117dup),M c.11763C>A (p.Tyr3921Stop)P | RRM | | | | | | | No Muscle Biopsy Performed |
| CC | c.2275A>G (p.Asn759Asp),P c.14422\_14424delTTC (p.Phe4808del)M | CCD | + | | | + | | | Increased variation in fiber size. On EM: disruption of myofibrillar apparatus |
