# Supplementary material for: Genotype-phenotype correlations in recessive RYR1-related myopathies
Source: Orphanet J Rare Dis. 2013 Aug 6;8:117. doi: 10.1186/1750-1172-8-117 (PMC3751094; doi:10.1186/1750-1172-8-117)
Supplement: Additional file 7: Table S7 — Clinical characteristics of newly reported dominant RYR1 mutations. Severity scores based on criteria listed in Additional file 3: Table S3. Polyphen2 scores are also included for all novel missense mutations (Probably Damaging (PRD)) and the severity (scale 0–1, 0 indicating least severe, 1 indicating most severe). ^ indicates that the silent mutation is predicted to create a new splice donor site resulting in a 25 amino acid in-frame deletion. Previously reported mutations: aLynch, et al., 1999, bDavis, et al., 2003, cManning, et al., 1998, dChamley, et al., 2000, eMonnier, et al., 2001, fDavis, et al., 2002). Origin of the mutation is designated M for maternal, P for paternal, or D for de novo. Abbreviations: Patient ID (ID), siblings (B&C), diagnosis (DX), central core disease (CCD), RYR1-related myopathy (RRM), multimincore disease (MmD), first year of life (FYOL), Weakness: proximal (P), distal (D), facial (F), neck (N); rigid spine (RS), ophthalmoparesis (OPH), respiratory distress (RD), ventilator (Vent), feeding difficulties (FD), malignant hyperthermia (MH), creatine kinase (CK). [file 1750-1172-8-117-S7.pptx]

## Slide 1
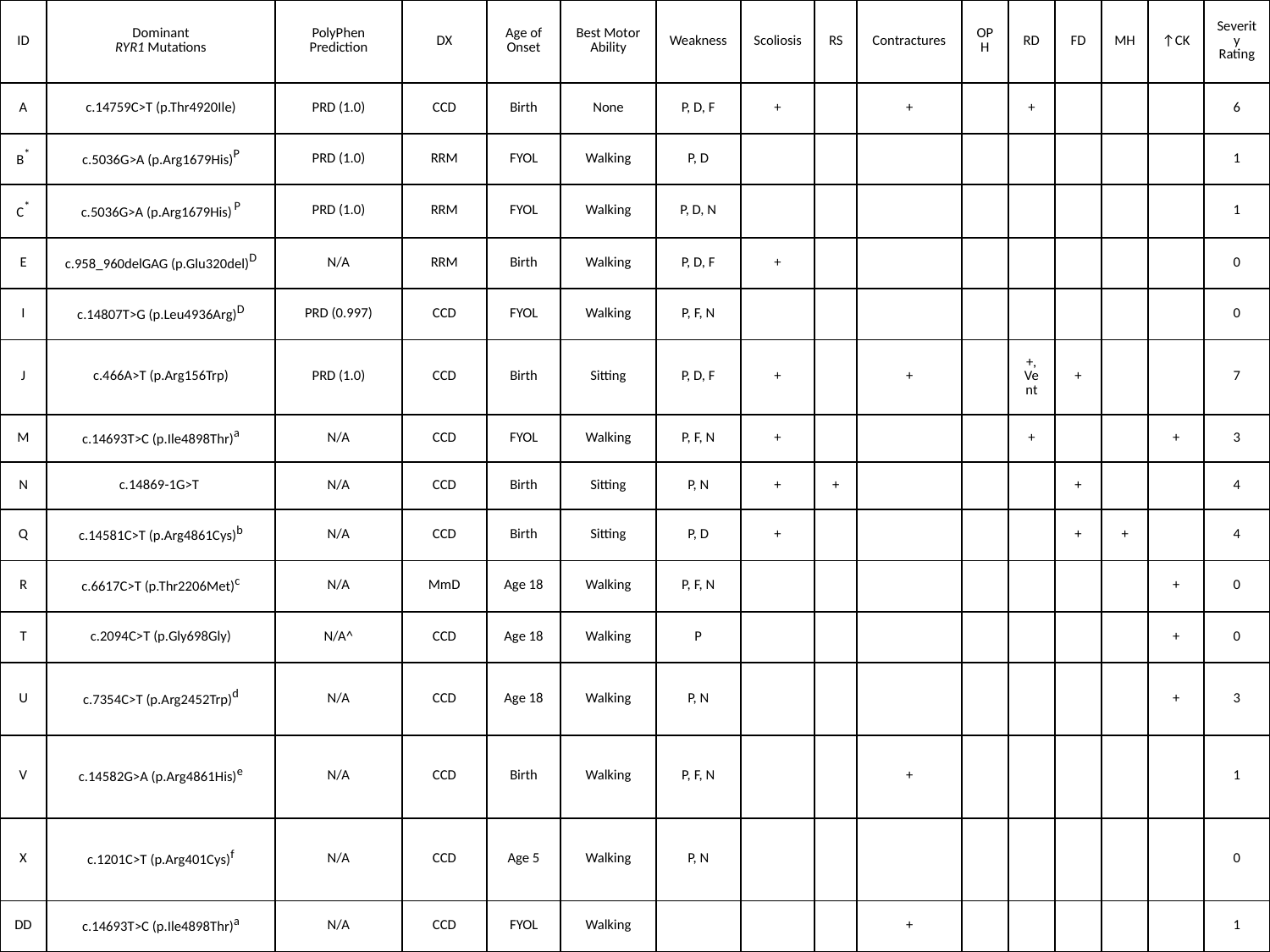

| ID | Dominant RYR1 Mutations | PolyPhen Prediction | DX | Age of Onset | Best Motor Ability | Weakness | Scoliosis | RS | Contractures | OPH | RD | FD | MH | ↑CK | Severity Rating |
| --- | --- | --- | --- | --- | --- | --- | --- | --- | --- | --- | --- | --- | --- | --- | --- |
| A | c.14759C>T (p.Thr4920Ile) | PRD (1.0) | CCD | Birth | None | P, D, F | + | | + | | + | | | | 6 |
| B\* | c.5036G>A (p.Arg1679His)P | PRD (1.0) | RRM | FYOL | Walking | P, D | | | | | | | | | 1 |
| C\* | c.5036G>A (p.Arg1679His) P | PRD (1.0) | RRM | FYOL | Walking | P, D, N | | | | | | | | | 1 |
| E | c.958\_960delGAG (p.Glu320del)D | N/A | RRM | Birth | Walking | P, D, F | + | | | | | | | | 0 |
| I | c.14807T>G (p.Leu4936Arg)D | PRD (0.997) | CCD | FYOL | Walking | P, F, N | | | | | | | | | 0 |
| J | c.466A>T (p.Arg156Trp) | PRD (1.0) | CCD | Birth | Sitting | P, D, F | + | | + | | +, Vent | + | | | 7 |
| M | c.14693T>C (p.Ile4898Thr)a | N/A | CCD | FYOL | Walking | P, F, N | + | | | | + | | | + | 3 |
| N | c.14869-1G>T | N/A | CCD | Birth | Sitting | P, N | + | + | | | | + | | | 4 |
| Q | c.14581C>T (p.Arg4861Cys)b | N/A | CCD | Birth | Sitting | P, D | + | | | | | + | + | | 4 |
| R | c.6617C>T (p.Thr2206Met)c | N/A | MmD | Age 18 | Walking | P, F, N | | | | | | | | + | 0 |
| T | c.2094C>T (p.Gly698Gly) | N/A^ | CCD | Age 18 | Walking | P | | | | | | | | + | 0 |
| U | c.7354C>T (p.Arg2452Trp)d | N/A | CCD | Age 18 | Walking | P, N | | | | | | | | + | 3 |
| V | c.14582G>A (p.Arg4861His)e | N/A | CCD | Birth | Walking | P, F, N | | | + | | | | | | 1 |
| X | c.1201C>T (p.Arg401Cys)f | N/A | CCD | Age 5 | Walking | P, N | | | | | | | | | 0 |
| DD | c.14693T>C (p.Ile4898Thr)a | N/A | CCD | FYOL | Walking | | | | + | | | | | | 1 |
