# Supplementary material for: Genotype-phenotype correlations in recessive RYR1-related myopathies
Source: Orphanet J Rare Dis. 2013 Aug 6;8:117. doi: 10.1186/1750-1172-8-117 (PMC3751094; doi:10.1186/1750-1172-8-117)
Supplement: Additional file 8: Table S8 — Hispathologic findings of newly reported dominant RYR1 mutations. Previously reported mutations: aLynch, et al., 1999, bDavis, et al., 2003, cManning, et al., 1998, dChamley, et al., 2000, eMonnier, et al., 2001, fDavis, et al., 2002) [8,36-39,44]. Origin of the mutation is designated M for maternal, P for paternal, or D for de novo. Abbreviations: Patient ID (ID), siblings (B&C), diagnosis (DX), central core disease (CCD), RYR1-related myopathy (RRM), multimincore disease (MmD), central cores (CC), minicores (MC), internalized nuclei (IN), central nuclei (CN). [file 1750-1172-8-117-S8.pptx]

## Slide 1
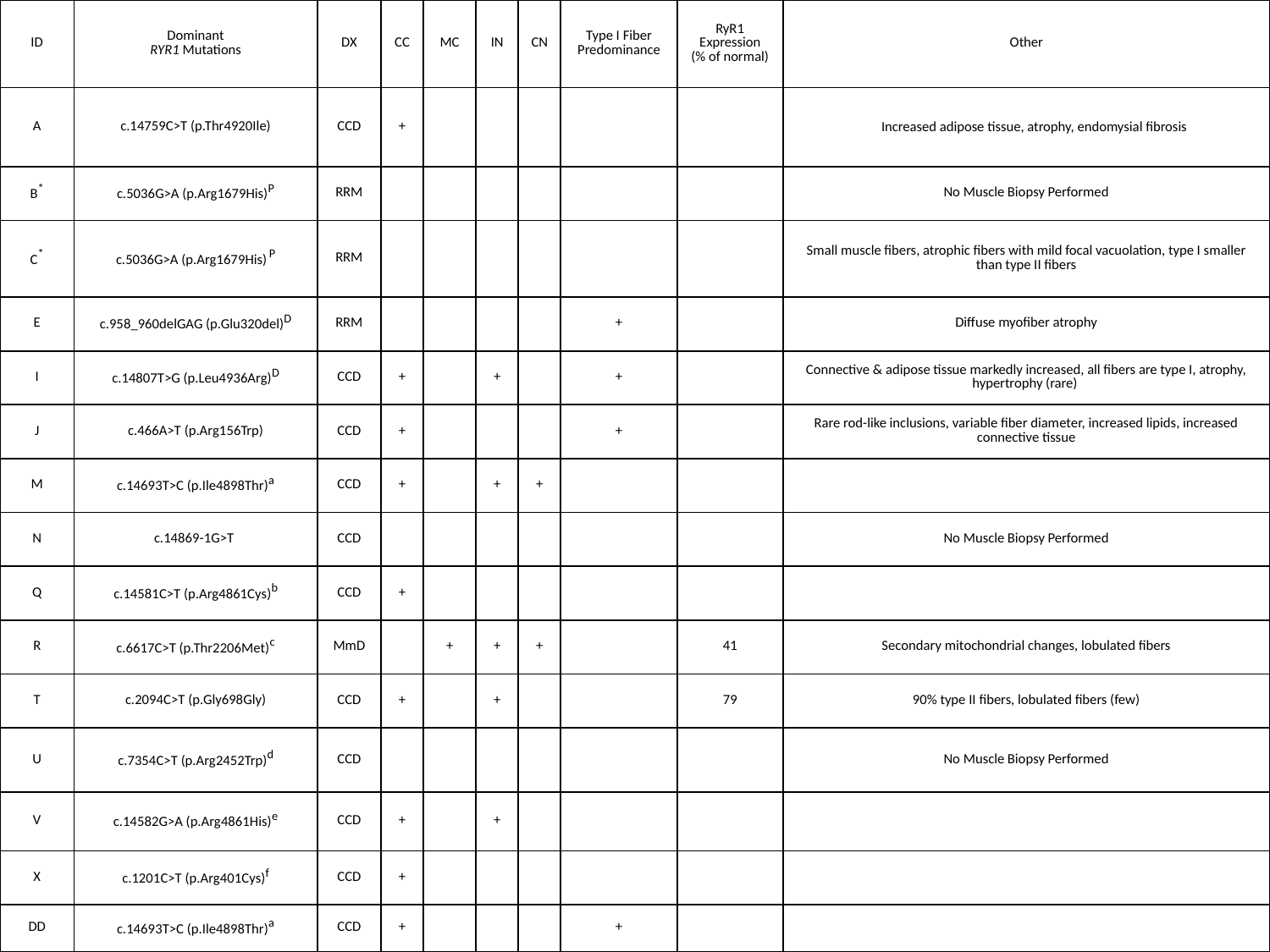

| ID | Dominant RYR1 Mutations | DX | CC | MC | IN | CN | Type I Fiber Predominance | RyR1 Expression (% of normal) | Other |
| --- | --- | --- | --- | --- | --- | --- | --- | --- | --- |
| A | c.14759C>T (p.Thr4920Ile) | CCD | + | | | | | | Increased adipose tissue, atrophy, endomysial fibrosis |
| B\* | c.5036G>A (p.Arg1679His)P | RRM | | | | | | | No Muscle Biopsy Performed |
| C\* | c.5036G>A (p.Arg1679His) P | RRM | | | | | | | Small muscle fibers, atrophic fibers with mild focal vacuolation, type I smaller than type II fibers |
| E | c.958\_960delGAG (p.Glu320del)D | RRM | | | | | + | | Diffuse myofiber atrophy |
| I | c.14807T>G (p.Leu4936Arg)D | CCD | + | | + | | + | | Connective & adipose tissue markedly increased, all fibers are type I, atrophy, hypertrophy (rare) |
| J | c.466A>T (p.Arg156Trp) | CCD | + | | | | + | | Rare rod-like inclusions, variable fiber diameter, increased lipids, increased connective tissue |
| M | c.14693T>C (p.Ile4898Thr)a | CCD | + | | + | + | | | |
| N | c.14869-1G>T | CCD | | | | | | | No Muscle Biopsy Performed |
| Q | c.14581C>T (p.Arg4861Cys)b | CCD | + | | | | | | |
| R | c.6617C>T (p.Thr2206Met)c | MmD | | + | + | + | | 41 | Secondary mitochondrial changes, lobulated fibers |
| T | c.2094C>T (p.Gly698Gly) | CCD | + | | + | | | 79 | 90% type II fibers, lobulated fibers (few) |
| U | c.7354C>T (p.Arg2452Trp)d | CCD | | | | | | | No Muscle Biopsy Performed |
| V | c.14582G>A (p.Arg4861His)e | CCD | + | | + | | | | |
| X | c.1201C>T (p.Arg401Cys)f | CCD | + | | | | | | |
| DD | c.14693T>C (p.Ile4898Thr)a | CCD | + | | | | + | | |
